# Comparing quantile regression methods for probabilistic forecasting of NO_2_ pollution levels

**DOI:** 10.1038/s41598-021-90063-3

**Published:** 2021-06-02

**Authors:** Sebastien Pérez Vasseur, José L. Aznarte

**Affiliations:** Artificial Intelligence Department, Universidad Nacional de Educación a Distancia — UNED, c/Juan del Rosal, 16, Madrid, Spain

**Keywords:** Environmental sciences, Mathematics and computing

## Abstract

High concentration episodes for NO2 are increasingly dealt with by authorities through traffic restrictions which are activated when air quality deteriorates beyond certain thresholds. Foreseeing the probability that pollutant concentrations reach those thresholds becomes thus a necessity. Probabilistic forecasting, as oposed to point-forecasting, is a family of techniques that allow for the prediction of the expected distribution function instead of a single future value. In the case of NO_2_, it allows for the calculation of future chances of exceeding thresholds and to detect pollution peaks. However, there is a lack of comparative studies for probabilistic models in the field of air pollution. In this work, we thoroughly compared 10 state of the art quantile regression models, using them to predict the distribution of NO_2_ concentrations in a urban location for a set of forecasting horizons (up to 60 hours into the future). Instead of using directly the quantiles, we derived from them the parameters of a predicted distribution, rendering this method semi-parametric. Amongst the models tested, quantile gradient boosted trees show the best performance, yielding the best results for both expected point value and full distribution. However, we found the simpler quantile *k*-nearest neighbors combined with a linear regression provided similar results with much lower training time and complexity.

## Introduction

Pollution has become a worrying issue in cities due to its adverse effects on health and the increase in pollutant concentrations, mainly due to human activity (traffic, heating systems...)^1^. In order to take preventive steps to maintain air quality, forecasting the evolution of pollution levels becomes a useful tool for decision makers: detecting pollution peaks beforehand could give cities enough time to take and communicate effective measures.

Multiple research papers have focused on this issue and have dealt with the prediction of air quality. Bai et al.^2^ describe the state of the art in this exercise and collect a range of diverse solutions applied to this problem.

However, the prediction of the expected value of pollution concentrations through point-forecasting does not provide enough information about the likelihood of the pollutant levels reaching a certain threshold. Indeed, we have an estimate but we usually do not have a description of the confidence of the model nor the uncertainty in the predictions. Therefore, it is difficult to estimate the probability of the pollutant reaching above a certain threshold.

The reason this probability estimation is so important is because the measures taken by cities to limit pollution (for example, limiting traffic) impact the daily routines of citizens and prove themselves to be quite unpopular. Therefore, local governments need to have an estimation of the confidence in the prediction to safely engage in those preventive measures.

As noted by Hothorn et al.^3^, the real objective in a regression analysis is to find the full conditional distribution of the target variable: in our case, the distribution of the concentration of the pollutants. Indeed, this full distribution gives an idea of the uncertainty of our predictions and can be useful to forecast the probability of the signal being above a certain threshold. For example, in the city of Madrid, hourly NO_2_ concentrations in the air are considered to be harmful from 180 $\upmu g m^{-3}$.

Previous research on the same dataset has already shown the usefulness of probabilistic forecasting for NO_2_ levels^4^, establishing the advantages of the approach and focusing on 1 h-ahead predictions with a single model (quantile random forests). We hereby extend that work by implementing other six models) quantile linear regression, quantile *k*-nearest neighbours, quantile gradient boosted trees, neural networks, distributional random forests, natural gradient boosting) and by using them for a wide set of forecasting horizons (up to 60 hours). Inspiration is drawn on some of the best approaches from the GEFCom forecasting competition^5,6^.

Furthermore, improving over these approaches, we also present a novel method to apply statistical inference to the output of the models. This method aknowledges the fact that the results show linear dependences between the predictors and the target, which slightly benefits linear models over nonlinear ones. By combining a linear model with nonlinear probabilistic modelling of its residuals we obtain optimized versions of the standard models.

## Probabilistic forecasting with quantile regression

The prediction from most regression models is a point estimate of the conditional mean of a dependent variable, or response, given a set of independent variables or predictors. However, the conditional mean does not provide a complete summary of the distribution, so in order to estimate the associated uncertainty, quantiles are in order. Then the median or 50 percentile can serve as a prediction of the center and for example, the 95 percentile represents the value of the response with 95% of the predicted points below. Recent advances in computing have inducted the development of regression models for predicting given quantiles of the conditional distribution. The technique is called quantile regression (QR) and was first proposed by Koenker in 1978^7^ based on the intuitions of the astronomer and polymath Rudjer Boscovich in the 18th century. Elaborating from the same concept of estimating conditional quantiles from different perspectives, several statistical and CI models that implement this technique have been developed: from the original linear proposal to multiple or additive regression, neural networks, support vector machines, random forests etc.

Quantile regression has gained an increasing attention from very different scientific disciplines^8^, including financial and economic applications^9^, medical applications^10^, wind power forecasting^11^, electric load forecasting^12^, environmental modelling^13^ and meteorological modelling^14^ (these references are just examples and the list is not exhaustive). To our knowledge, despite its success in other areas, quantile regression has not been applied in the framework of air quality, with the exception of^15^.

Thus, as we can estimate an arbitrary quantile and forecast its values, we can also estimate the full conditional distribution and fit it to a known distribution. This will entail us to the results presented in section “Results and discussion”.

Among the growing array of methods that allow to estimate and forecast data-driven conditional quantiles, in this study we have chosen to compare linear regression, *k*-nearest neighbors, random forests and gradient boosted trees. We took the point-estimate version of those models and converted them to their quantile or probabilistic counterparts. We can therefore compare each model not only on the accuracy of their point estimation but on the confidence of each model.

We will compare the different algorithms through the Root Mean Squared Error (RMSE), Mean Absolute Error (MAE) and bias for the quantile 50 and the continuous ranked probability score (CRPS) metric for the forecast distribution. As described by Gneiting et al.^16^, CRPS is a measure of the squared difference between the forecast cumulative distribution function (CDF) and the empirical CDF of the observation.

## Data description and experimental design

### Nitrogen dioxide

**Figure 1:**
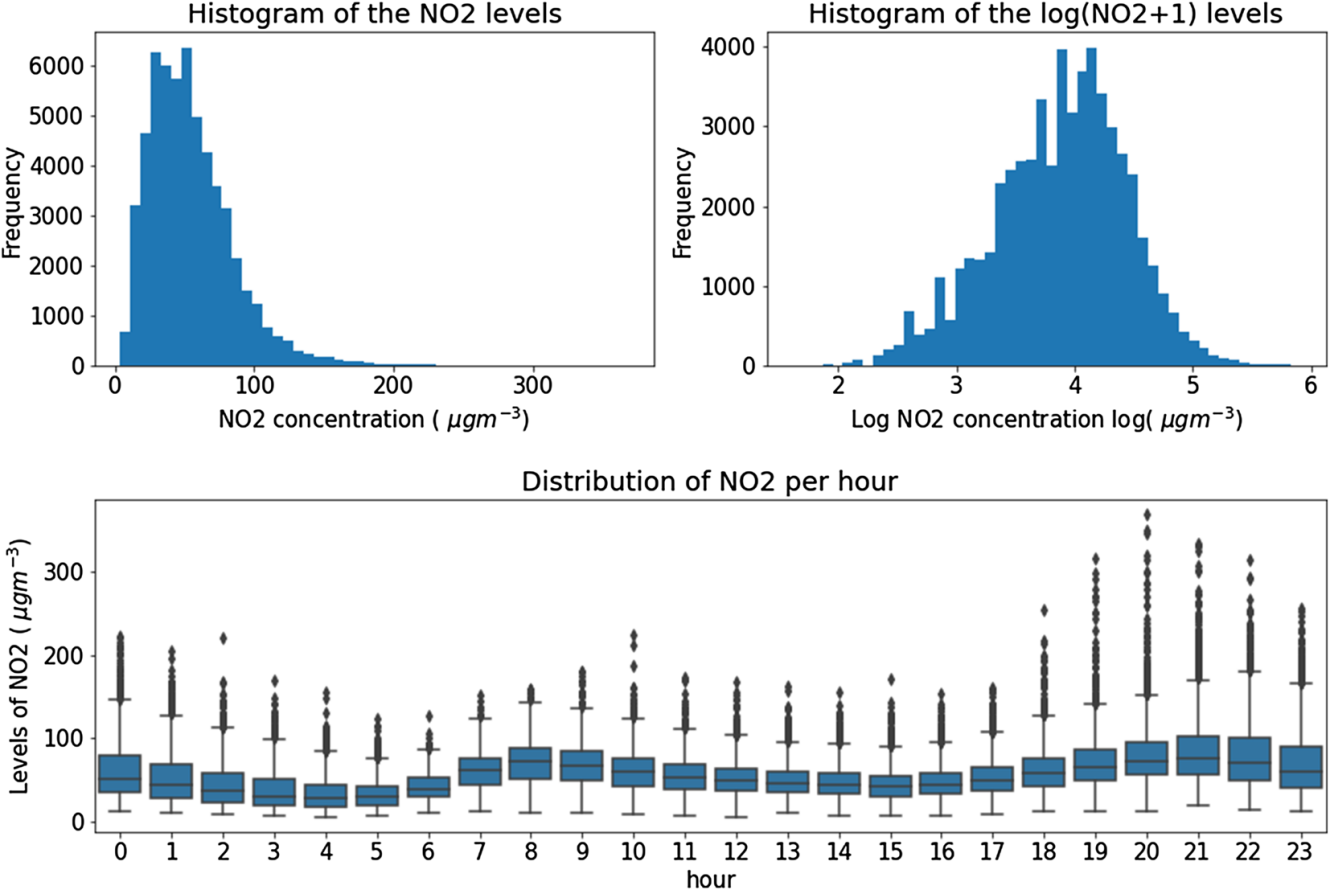
Distribution of logarithmic NO_2_ and distribution of NO_2_ per hour.

The city of Madrid has an air quality monitoring system composed by 24 stations which capture hourly data for NO_2_. For this study, we have selected one of the stations with higher average leves: *Escuelas Aguirre* station (code 28079008).

As we can see in Fig. 1, the shape of the histogram approaches the one from a lognormal distribution and therefore we transformed to the logarithm of the values. This has 2 positive effects: it reduces the tail of the distribution which will enable better quantile estimation and it reduces the skewness of the distribution which helps with linear models like the linear quantile regression.

The time series for this station consists of hourly measured values of the concentrations of NO_2_ from 01/01/2013 to 31/12/2019. These values exhibit a clear intraday pattern, in which the higher values are located in two peaks around the morning and evening (with highest average value around 19 h) while the nightly hours (from 00 h to 05 h) have lower average concentrations. Not only are the values higher at those hours, but also the variance is, as we can see in Fig. 1.

In order to analyze the seasonality of the signal, we extract the 5 main factors from the Fourier transform. Those correspond to the main repetitive patterns found on the series, and can be seen clearly from the first 3000 components. The series shows certain seasonality for 12-h, 24-h, 1 week (168-h) and one year. Therefore, we will create, and use as inputs for the models, the output of periodic functions (cosine and sine) whose frequency is equal to the ones stated above. This will enable the machine learning models to learn the seasonality of our time series.

As is common when forecasting with machine learning models, we exploit the inertia of the modelled series by adding lagged variables to the inputs. Of course, in doing so, we are limited by the horizon of the prediction and by the ’curse of dimensionality’, which implies keeping a limited number of features as input. In our case, the inertia of the series will be modeled by lagged values from the inmediate past (hours before) and, based on the seasonal analysis: 1–5 h before and every 11–13 h up to 9 days before.

### Ozone

The same station that records Nitrogen dioxide also records the levels of ozone (O_3_). It is known that ozone and Nitrogen dioxide are related by chemical reactions occurring in the atmosphere in the presence of sunlight, especially of the UV spectrum. Thus, we will also add lagged values of O_3_ as inputs to our models.

### ECMWF numerical pollution prediction

The European Centre for Medium-Range Weather Forecasts (ECMWF) implements the Copernicus Atmosphere Monitoring Service. This service delivers a daily production of near-real-time European air quality analyses and forecasts with a multi-model ensemble system. Although these forecasts are a very good starting point, the resolution of the model is 10km and hence it is not expected to be capable of modelling the local urban effects of the NO_2_ series under study.

### Calendar variables

As NO_2_ levels are clearly be linked to human activity, we will also flag the hours belonging to a specific type of day. Days could be classified as bank holidays, heavy traffic days (for example, return from holidays), school holidays...We will also use as inputs to the models past values of this variables (1, 2 and 7 days before).

### Experimental design

**Figure 2:**
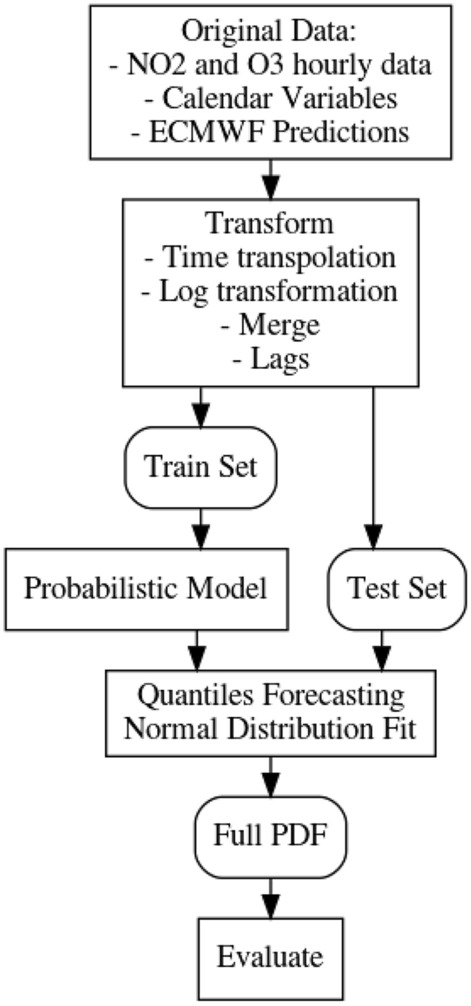
Data flow of the experiments.

As a summary, we use the following predictors: NO_2_ measures lagged 1–5 h and every 11–13 h up to 9 days before, O_3_ levels lagged every 24 h up to 4 days before, calendar variables lagged 1, 2 and 7 days before, ECMWF predictions and seasonal features extracted from the Fourier analysis. This amounts to a total of 102 independent variables.

When performing the experiments, first we aligned and gathered all the hourly time series: NO_2_, O_3_, ECMWF and calendar variables. Then we transformed the signal levels and then we added the lagged values and a seasonal time series with the main periods of the NO_2_ time series.

Once all this process is finalized, we train the following probabilistic models: quantile random forests (QRF), *k*-nearest neighbors (QKNN), quantile linear regression (QLR) and quantile gradient boosting (QGB), Multilayer perceptron (MLP), Distributional random forests (DT) and NGBoost (NGBOOST). Figure 2 shows the data flow in the experimental design. All the hyperparameters of the models have been estimated through grid search on a validation set. In section “Probabilistic models” we provide more information on each of the models.

Concerning cross-validation, there are several accepted methods to separate the train and test set which produce correct estimations of the error^17^: We can approximate the error of a model through a cross-validated evaluation as long as we removed from each test set the correlated samples to their respective training dataset. We chose a cross-validation with 5 splits for time execution reasons. We will always test with predictions done at 10:00, as this is the time the forecast is done in the operational setting, as the data is first available at that time.

We want to forecast the full distribution of NO_2_ levels for the next 60 h and therefore we will train and evaluate the models for each hour (60 horizons).

After forecasting the quantiles, we will fit them to a normal distribution. Fitting a normal distribution to the predicted quantiles and then generating the percentiles for that fitted distribution has several advantages. It enables the calculation of more percentiles from a small number of them. It also helps estimating the upper tail of the distribution, in spite of the low probability for those values.

We will evaluate the predicted 50 percentile through standard evaluation metrics (RMSE and bias), and the predicted distribution through the CRPS.

We will perform this evaluation for each of the models and each of the horizons.

### Probabilistic models

As stated above, we will compare seven different probabilistic models, which are briefly described below for reference. We will provide alongside the models the abreviation we will use for each of them throughout this article. Also we are adapting point-estimation algorithms to their probabilistic counterparts. This allows us to see the uncertainty these models have. Indeed, metrics like RMSE and Bias are linked to the confidence of the models for point estimation but the predicted CDF is much better at describing it. This also increases the interpretability of those models. Therefore the implementations we described can have applications beyond this forecast exercise.

#### Quantile linear regression (QLR)

As shown in^7^, we can apply linear regression with a modified cost function in order to predict the quantiles of the dependent variable. Given a set of vectors $(x_i, y_i)$, in the usual point forecasting approach we are usually interested in the prediction $\hat{y}(x) = \alpha _0 + \alpha _1 x$ which minimizes the mean squared error,

1 $$\begin{aligned} E = \frac{1}{n} \sum ^n_i \epsilon _i = \frac{1}{n} \sum ^n_i [ y_i - (\alpha _0 + \alpha _1 x) ]^2. \end{aligned}$$

This prediction is the conditional sample mean of *y* given *x*, that is, $\hat{y}(x) = \hat{\alpha }_0 + \hat{\alpha }_1 x$, or the location of the conditional distribution. But we could be interested in estimating the conditional median (i.e., the 0.5 quantile) instead of the mean, in which case we should find the prediction $\hat{y}(x)$ which minimizes the mean absolute error,

2 $$\begin{aligned} E = \frac{1}{n} \sum ^n_i \epsilon _i = \frac{1}{n} \sum ^n_i | y_i - (\alpha _0 + \alpha _1 x) |. \end{aligned}$$

The fact is that, apart from the 0.5 quantile, it is possible to estimate any other given quantile $\tau$. In that case, instead of (2), we could minimize

3 $$\begin{aligned} E= \frac{1}{n} \sum ^n_i f( y_i - (\alpha _0 + \alpha _1 x)) \end{aligned}$$

where

4 $$\begin{aligned} f(y-q) = \left\{ \begin{array}{l l} \tau (y-q) &{} \quad \text{ if } y \ge q\\ (1-\tau ) (q-y) &{} \quad \text{ if } y < q\\ \end{array} \right. , \end{aligned}$$

with $\tau \in (0,1)$. Equation (3) represents the median when $\tau =0.5$ and the $\tau$-th quantile in any other case.

We will train 5 linear regression models to predict 5 percentiles of the signal. Those 5 percentiles will enable us to calculate the mean and standard deviation of a normal distribution. This normal distribution will provide the predicted quantiles.

#### Quantile *k*-nearest neighbors (QKNN)

We will use the probabilistic *k*-nearest neighbors algorithm as described in^6^. This algorithm is based on the standard *k* nearest neighbor, where instead of calculating the mean of the targets of the *k* nearest points to the input, it fits a normal distribution to the targets of those neighbors. We build the quantiles from the predicted normal distribution.

#### Quantile random forests (QRF)

Quantile random forests create probabilistic predictions out of the original observations. They work like the usual random forest, except that, in each tree, leafs do not contain a single value as a prediction but the target observations from the training set belonging to that leaf.

Then predictions are calculated by selecting the leafs in each tree corresponding to the input features and combining the weighted histograms in each tree out of the target observations in those leafs. For more information refer to^18^. We then fit a normal distribution to the predicted histogram to calculate the predicted quantiles.

#### Quantile gradient boosted trees (QGB)

Tree boosting^19^ is a successful machine learning technique that consist on growing trees based on the compromise of a cost function and a regularization function. This technique has already been used to predict air pollution, as documented by Lee et al.^20^. The cost function is usually used to forecast the mean of the signal. We will modify the cost function (im a similar way as in the quantile linear regression) to predict the quantiles of the target. Also, we will use the lightgbm implementation^21^ which provides lower training times and higher accuracy.

We will train 5 gradient boosted trees models to predict 5 percentiles of the NO_2_ signal. Then, we will use the percentiles to fit a normal distribution and calculate the predicted quantiles.

#### Multilayer perceptron

We will also test a multilayer perceptron (MLP)^22^ with 1 hidden layer. Like for quantile linear regression and quantile gradient boost, we will modify the cost function to predict the quantiles of the target.

We will therefore as well train 5 MLP to predict 5 percentiles of the NO_2_ signal and calculate the final quantiles through a normal distribution as for the quantile linear regression and quantile gradient boosted tree.

#### Distributional random forests

Based on the idea of GAMLSS, Distributional Forests^23^ extend the power of this semiparametric method by estimating the LSS (location, scale and shape) through the use of random forests, instead of using GAM’s. We will use the R implementation of this method from the package disttree.

#### NGBoost

NGBoost^24^ uses the natural gradient instead of the standard gradient to build a boosted ensemble learner that predicts directly the parameters of the distribution of the target variable. Natural Gradients are presented as offering better stability and robustness than normal gradients.

#### Probabilistic forecast of linear regression residuals

We suspect there are high linear dependencies between the input predictors and the target. We will then experiment on combining 3 of the models: quantile random forest, quantile *k*-nearest neighbor and quantile gradient boost with a linear regression.

We decided to train a linear regressor which predicts the NO_2_ values and then use the QRF, QKNN and QGB models to predict the full distribution of the residuals of that linear regression. We abbreviate both models respectively with QRFL, QKNNL and QGBL.

## Results and discussion

**Figure 3:**
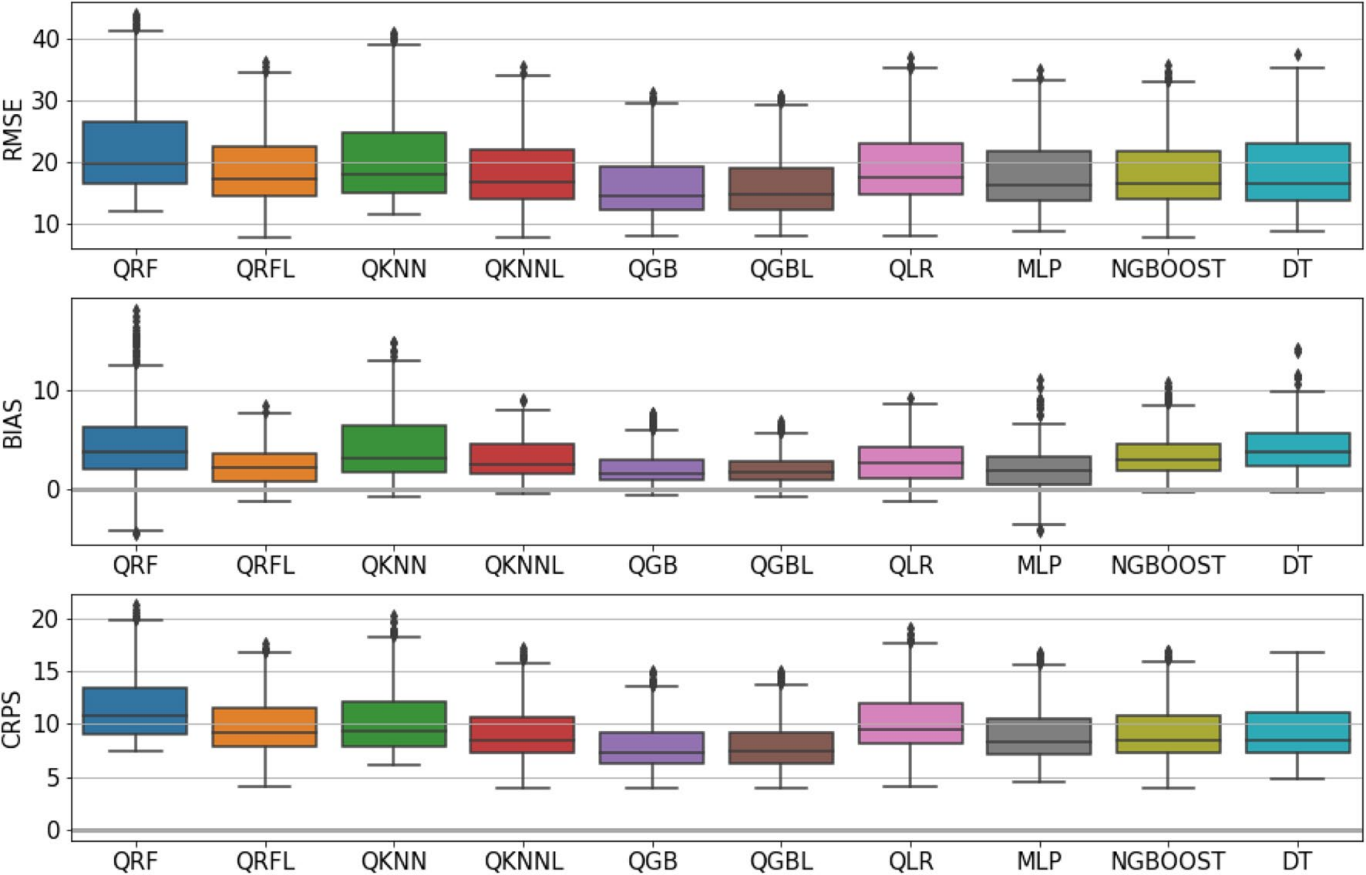
Boxplot of continuous ranked probability score, root mean squared error and bias of the different models for all horizons.

**Table 1:** Error measures for the proposed models.

method	RMSE	CRPS	bias	training time (s)
QRF	$\underset{(8.37)}{22.62}$	$\underset{(3.58)}{11.86}$	$\underset{(4.77)}{4.68}$	$\underset{(1.01)}{24.15}$
QRFL	$\underset{(6.52)}{19.12}$	$\underset{(3.03)}{9.97}$	$\underset{(2.0)}{2.32}$	$\underset{(0.36)}{8.71}$
QKNN	$\underset{(7.91)}{20.73}$	$\underset{(3.59)}{10.52}$	$\underset{(3.53)}{4.26}$	$\underset{(0.31)}{6.92}$
QKNNL	$\underset{(6.44)}{18.4}$	$\underset{(2.99)}{9.34}$	$\underset{(2.01)}{3.06}$	$\underset{(0.32)}{7.06}$
QGB	$\underset{(5.59)}{16.09}$	$\underset{(2.54)}{8.04}$	$\underset{(1.76)}{2.08}$	$\underset{(1.19)}{42.78}$
QGBL	$\underset{(5.55)}{16.14}$	$\underset{(2.55)}{8.1}$	$\underset{(1.55)}{1.95}$	$\underset{(1.04)}{42.22}$
QLR	$\underset{(6.71)}{19.44}$	$\underset{(3.31)}{10.38}$	$\underset{(2.16)}{2.77}$	$\underset{(0.53)}{9.68}$
MLP	$\underset{(6.04)}{18.16}$	$\underset{(2.85)}{9.27}$	$\underset{(2.49)}{1.86}$	$\underset{(7.71)}{73.08}$
NGBOOST	$\underset{(6.43)}{18.52}$	$\underset{(2.96)}{9.4}$	$\underset{(2.32)}{3.42}$	$\underset{(14.2)}{180.86}$
DT	$\underset{(6.77)}{18.69}$	$\underset{(2.99)}{9.45}$	$\underset{(2.84)}{4.27}$	$\underset{(12.29)}{289.81}$

**Figure 4:**
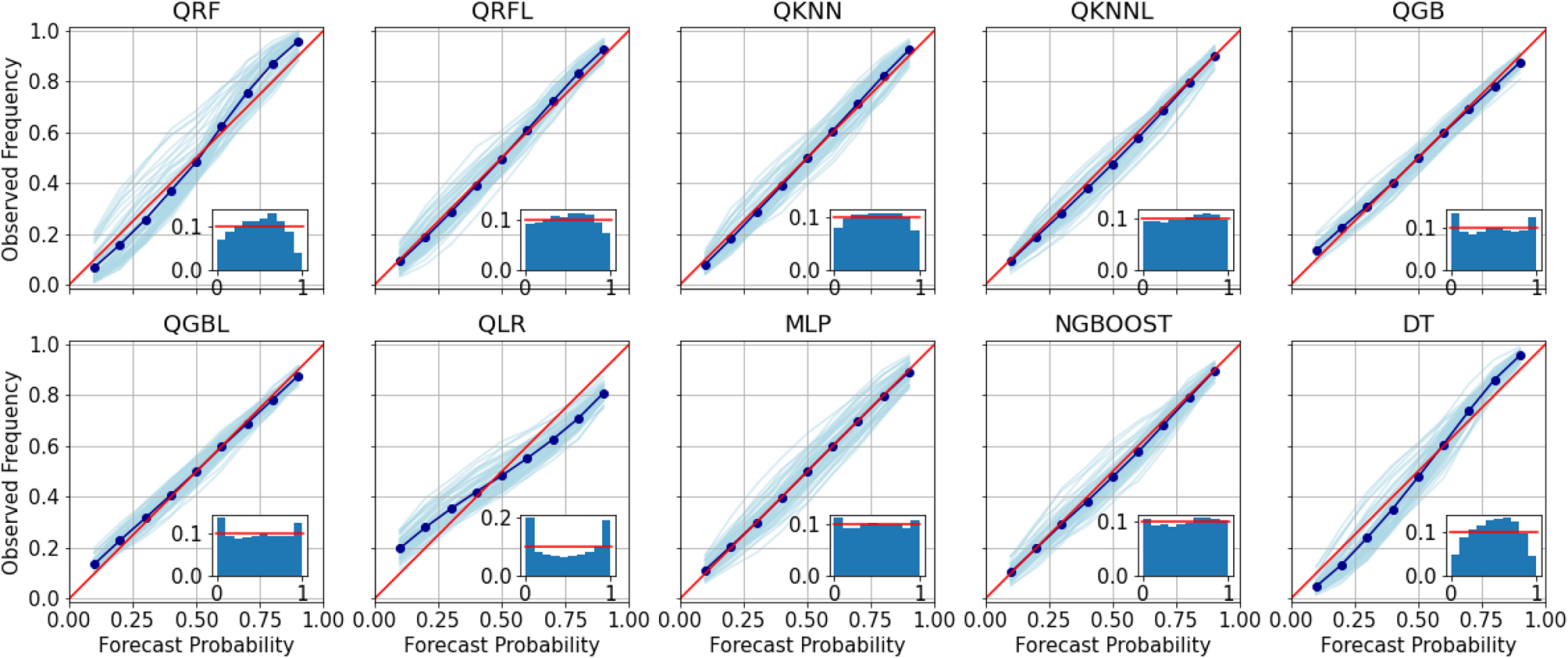
Average reliability and sharpness of the different models across all horizons. The dim blue lines correspond to the different horizons.

Figure 3 shows the different metrics for each model across all the horizons. First, we see how quantile gradient boosted trees (QGB) outperforms the other models and displays better scores for all metrics. The additive nature of QGB is behind these results.

Quantile random forests and quantile *k*-nearest neighbors underperform compared to the other models, showing a bias which is clearly higher compared to the others. The main reason for this can be attributed to the highly linear dependence of the data. This also explains the relatively good results of the linear model (QLR). However, the linear model underperforms when compared to other models, as it is unable to learn the non-linear relationships between the predictors and the target.

Regarding the models predicting the residuals of a linear regression, QKNNL and QRFL showcase good performance, which is remarkable for QKNNL due to the simplicity of this approach. The KNNL offers similar results to QGB with lower complexity and lower training times. On the other hand, QGBL brings no real benefit compared to QGB, since QGB can already integrate the linear component.

Distributional forests (DT) and natural gradient boosted trees (NGB) present relatively good results but are still outperformed by QGB. Natural gradients bring no real benefit in this case.

Finally, MLP has good bias results but underperforms against the other methods.

As stated earlier, the NO_2_ levels follow a lognormal distribution and thus it seems that it is better modeled with a multiplicative model (since different causes multiply the levels of pollution), therefore the logarithm of the NO_2_ levels is better forecast with an additive model. This would explain why quantile gradient boosted trees consistently outperforms the other models: it can naturally add the nonlinear effects.

Table 1 displays the mean and standard deviation of CRPS of the different models. The table shows again the good performance of the quantile gradient boosted trees model for CRPS. The table also shows the training times of the different models. QGB appears as having a good compromise between low training times and metrics. QKNNL offers good performance with really low training times.

As stated above, CRPS is a good summary of the performance of probabilistic models. Notwithstanding, the reliability and sharpness graphs are known to be useful at estimating how the observed values are positioned in the distributions. Figure 4 features the reliability and sharpness of the different models.

The sharpness is given by the distribution of the forecast probabilities (small histogram overimposed in the graphs). NGBOOST, MLP and QKNNL display the best results as their histograms are almost flat.

For QRF, QRFL, QKNN and DT, we see fewer observations on the sides of the predicted distributions. This means the variance of the predicted variance is too high and the predictions have too much uncertainty. It is specially noticeable for QRF and DT.

On the contrary, QGB, QGBL and QLR seem more balanced but display high values at both sides of the sharpness curve. If we consider the distribution to be a Gaussian distribution, this means the forecast distributions tend to have a too small standard deviation and are too narrow. Therefore, the forecast probability of some levels of NO_2_ is too small compared to the observed one. QGBL and MLP also display this behaviour. Since, in this framework, it is common that the main interest lies in predicting the higher tail of the distribution (representing peaks of pollution), this is not considered to be a critical problem in this case. Finally, we see again the QKNNL model performing similarly to the QGB model, offering a simpler alternative to QGB.

Reliability is shown in the main charts of Fig. 4. The light blue lines represent each of the reliability diagrams for each horizon, while the dark blue line shows the average reliability diagram for a single model over all the horizons. This let us see how stable is the reliability per horizon.

It is noticeable that QGBL and QKNNL show the best results both in terms of the average diagram and the dispersion of the different diagrams per horizon. MLP and NGBOOST have a good average line but also display a high variability for each horizon. On the other hand, QRF, QRFL, QKNN and DT also show a high variability and again display the high uncertainty of their predictions, which is seen in the extremes of the reliability diagram. On the contrary, QGB and QLR show the opposite behaviour with narrow predicted variances.

We also see that NGBOOST and QKNNL seem to predict better the tail of the distributions (quantile 90). This is relevant as we are interested in the higher values of NO_2_ pollution levels.

In order to compare the result of the 10 methods, we will use advanced non parametric tests as described by García et al.^25^. The Quade test will calculate the rank of all the algorithms and check if they are significantly different from the mean rank. We have 60 different datasets, each one corresponding to a horizon where we have performed a fivefold crossvalidation and kept the mean of the results. The rank is calculated not only based on the results in the different datasets but also on the importance of the dataset (based on the variance of results), this being the main difference with a Friedmann test. The result of the Quade test gives a p-value of 1.11e–16 which rejects the null hypothesis that the methods have similar performance and thus we can use the ranks to classify the different methods. Table 2 shows the ranks of the Quade test.

**Table 2:** Average rankings of the algorithms (Quade).

Algorithm	Ranking
QRF	7.354393
QRFL	4.888386
QKNN	6.522557
QKNNL	3.379697
QGB	0.967290
QGBL	1.297199
QLR	5.791688
MLP	2.883587
NGBOOST	3.442853
DT	3.921512

## Conclusions

After extracting and processing the data from one of the pollution stations in Madrid, we have compared 10 different models to build a probabilistic forecast of the levels of NO_2_ for up to 60 hours into the future. We have evaluated our models through the forecast quantile 50 and the full forecast distribution. We have also adapted the predicted quantiles to fit a normal distribution.

We have observed a linear dependence between the target and the features. For this reason, the linear quantile regression has performed well compared to random forests and *k*-nearest neighbors. However, the multiplicative nature of the levels of NO_2_ and the nonlinear dependence between target and features have lead to better results for the gradient boosted trees which has outperformed all the other models in all metrics.

However, we have shown how quantile random forest and quantile *k*-nearest neighbors could be used to improve the results of a linear model when nested to model the full distribution of the residuals of a linear regression. Those models, specially the *k*-nearest neighbor are easier to train, so they become worthy and simpler alternatives to the gradient boosted trees.

These results are especially useful for practitioners that need to choose a probabilistic model for air quality time series and other similar problems with a strong anthropogenic component.
